# Artificial intelligence in patient education: evaluating large language models for understanding rheumatology literature

**DOI:** 10.3389/fdgth.2025.1623399

**Published:** 2025-10-15

**Authors:** Claudia Mendoza-Pinto, Pamela Munguía-Realpozo, Ivet Etchegaray-Morales, Edith Ramírez-Lara, Juan Carlos Solis-Poblano, Máximo Alejandro García-Flores, Jorge Ayón-Aguilar

**Affiliations:** ^1^ Unidad de Enfermedades Reumáticas y Autoinmunes Sistémicas, Unidad Médica de Alta Especialidad- Centro de Investigación Biomédica de Oriente, Instituto Mexicano del Seguro Social, Puebla, México; ^2^ Departamento de Reumatología, Facultad de Medicina, Benemérita Universidad Autónoma de Puebla, Puebla, México; ^3^ Systemic Autoimmune Rheumatic Diseases Research Unit, Specialties Hospital Unidad Médica de Alta Especialidad, Instituto Mexicano del Seguro Social, Puebla, Mexico; ^4^ Department of Hematology, Specialties Hospital UMAE, Instituto Mexicano del Seguro Social, Puebla, Mexico; ^5^ Coordination of Health Research, Instituto Mexicano del Seguro Social, Puebla, Mexico

**Keywords:** rheumatology, health literacy, patient education, readability, large language models, ChatGPT, peer-reviewed literature, artificial intelligence

## Abstract

**Background:**

Inadequate health literacy hinders positive health outcomes, yet medical literature often exceeds the general population's comprehension level. While health authorities recommend patient materials be at a sixth-grade reading level, scientific articles typically require college-level proficiency. Large language models (LLMs) like ChatGPT show potential for simplifying complex text, possibly bridging this gap.

**Objective:**

This study evaluated the effectiveness of ChatGPT 4.0 in enhancing the readability of peer-reviewed rheumatology articles for layperson comprehension.

**Methods:**

Twelve open-access rheumatology articles authored by the senior investigators were included. Baseline readability was evaluated utilizing Flesch-Kincaid Grade Level (FKGL) and Simple Measure of Gobbledygook (SMOG) indices. Each article was processed by ChatGPT 4.0 with a prompt requesting simplification to a sixth-grade level. Two expert rheumatologists evaluated the generated summaries’ appropriateness (accuracy, absence of errors/omissions). Readability changes were analyzed using paired *t*-tests.

**Results:**

ChatGPT significantly improved readability (*P* < .0001), reducing the average reading level from approximately 15th grade (FKGL: 15.06, SMOG: 14.08) to 10th grade (FKGL: 10.52, SMOG: 9.48). The expert reviewers deemed the generated summaries appropriate and accurate. The average word count was significantly reduced from 3,517 to 446 words (*P* = 0.047).

**Conclusions:**

ChatGPT effectively lowered the reading complexity of specialized rheumatology literature, making it more accessible than the original publications. However, the achieved 10th-grade reading level still exceeds the recommended sixth-grade level for patient education materials. While LLMs are a promising tool, their output may require further refinement or expert review to meet optimal health literacy standards and ensure equitable patient understanding in rheumatology.

## Introduction

Health literacy is pivotal in enhancing individual agency, enabling informed choices regarding health matters, effective interaction with healthcare systems, and managing their well-being. Initially defined by basic reading and writing skills, the concept now encompasses higher-order abilities such as critical thinking, decision-making, and effective communication (1). Low health literacy has been consistently linked to adverse outcomes, including poor treatment adherence and increased mortality (2). Studies reveal that nearly half of U.S. adults—and a substantial portion of Europeans—struggle to comprehend health information, thereby contributing to disparities in healthcare access and utilization (3). In response, organizations such as the World Health Organization have advocated for improved health communication, developing literacy-friendly environments, and comprehensive policies to enhance health literacy. Despite significant research efforts, gaps remain in our understanding of how low health literacy exacerbates disparities and which interventions are most effective, underscoring the need for further exploration of screening methods, communication strategies, and the underlying causal mechanisms (4).

In rheumatology, health literacy plays a critical role in patient care by influencing disease management, treatment adherence, and overall health outcomes (5). For instance, approximately one in seven patients with rheumatoid arthritis (RA) may lack the skills required to engage in informed decision-making, potentially leading to suboptimal disease control (6). Limited health literacy in rheumatic conditions such as RA and systemic lupus erythematosus (SLE) has been associated with higher disease activity and reduced understanding of self-management strategies (5, 7). Patients with inadequate health literacy often encounter difficulties with complex treatment regimens, resulting in medication non-adherence and a reluctance to modify therapy when necessary (8). These issues are particularly pronounced among vulnerable groups—such as older adults, ethnic minorities, and individuals of lower socioeconomic status—who are more likely to experience limited health literacy (5). Consequently, these patients are at an increased risk of poorer outcomes and may have restricted access to advanced treatments, including biologic disease-modifying antirheumatic drugs (DMARDs) (8). Addressing health literacy through targeted patient education and tailored communication is crucial to improving patient engagement and optimizing treatment adherence in rheumatologic care.

The American Medical Association (AMA) and the National Institutes of Health (NIH) suggested that patient-directed health materials be written at a sixth- to eighth-grade reading level (9). This guideline is critical in the digital era since most U.S. population accesses health information online. Despite these recommendations, research shows that much online rheumatology-related health content, including materials produced by academic institutions, often exceeds the advised readability levels (10, 11). The advent of large language models (LLMs) such as ChatGPT has recently opened new avenues for enhancing the accessibility of complex health information (12, 13). Our study aimed to evaluate the usability and accuracy of ChatGPT in transforming technical rheumatology literature into patient-friendly materials that adhere to AMA and NIH readability guidelines.

## Methods

To perform this analysis, we selected 12 open-access articles on diverse rheumatology topics authored by the two researchers (CMP and PMR). These articles spanned different study designs, including retrospective studies and systematic reviews. We then evaluated their readability using two well-established tools: the Flesch-Kincaid Grade Level (FKGL) and the Simple Measure of Gobbledygook (SMOG). The FKGL and the SMOG have emerged as the two most frequently employed readability assessment tools. Both formulas integrate metrics, including total word count, sentence count, syllable count, and the number of polysyllabic words. An online calculator (*via* the readability formulas website) was utilized to compute these readability scores. We subsequently provided ChatGPT 4.0 with the prompt: “Could you please simplify the following text, sourced from a peer-reviewed scientific paper? The objective is to achieve a 6th-grade reading level to make it fully understandable for a general reader. I will supply the text”. Appropriateness was assessed according to criteria previously published by two rheumatologist researchers (CMP and PMR), the authors of the selected articles. The outputs were classified as “appropriate” or “inappropriate” based on the evaluators’ clinical experience and understanding of the relevant literature.

The criteria for an appropriate response required the accurate simplification of the full text without introducing any false or misleading information. In contrast, an inappropriate response either contained inaccurate content or included material not intended by the original study authors. In cases of disagreement between the two evaluators, an independent opinion was sought from a third fellowship-trained uveitis physician. Statistical analyses were performed using SPSS software (version 26.0). A paired-sample *t*-test compared the original texts’ average readability scores and word counts with those of the ChatGPT-generated responses in achieving the advised readability level equivalent onto the sixth grade. At the same time, descriptive statistics summarized the remaining data. Given the absence of human participants and their data in this investigation, obtaining informed consent was unnecessary.

## Results

The application of ChatGPT significantly improved the readability of scientific articles (*P* < .0001). The mean readability level was reduced from approximately a 15th-grade level in the original texts to a 10th-grade level in the generated outputs. This reduction was observed consistently across all analyzed study types. Furthermore, the outputs generated by ChatGPT were evaluated as appropriate, accurately reflecting the source material in a simplified format without substantive errors. A significant decrease in length was also noted, with mean word counts reducing from 3,517 (SD 843) in the original articles to 446 (SD 172) in the generated responses (*P* = 0.047). Detailed data regarding response readability, appropriateness, original readability metrics, and study types are presented in Table 1.

**Table 1:** Overview of readability metrics and appropriateness for selected open-access research articles.

Title	Type	Original FKGL^a^	ChatGPT FKGL	Original SMOG^b^	ChatGPT SMOG	Appropriateness (Yes/No)
Risk of diabetes mellitus in systemic lupus erythematosus: systematic review and meta-analysis	Systematic Review	15.26	11.4	13.62	9.48	Yes
Predictors and prognostic factors influencing outcomes of anti-CD20 monoclonal antibodies in systemic lupus erythematosus: A systematic review update	Systematic Review	15.15	12.27	13.64	10.52	Yes
Burden of Other Musculoskeletal Disorders in Latin America and the Caribbean: Findings of Global Burden of Disease Study 2019	Epidemiological	15.11	11.66	14.2	10.26	Yes
SLICC-Frailty Index and Its Association with Low Bone Mineral Density and Vertebral Fractures in Women with Systemic Lupus Erythematosus	Retrospective	12.91	8.54	12.57	7.05	Yes
Trends in mortality in patients with systemic autoimmune rheumatic diseases (SARD) during the COVID-19 pandemic in Mexico	Epidemiological	12.84	11.14	12.98	10.75	Yes
Improving access to SLE therapies in low and middle-income countries	Review	18.26	11.56	16.41	9.7	Yes
Predicting progression from undifferentiated connective tissue disease to definite connective tissue disease: A systematic review and meta-analysis	Systematic Review	15.32	12.98	14.49	11.2	Yes
Temporal Trends in Mortality in Patients with Systemic Sclerosis in Public Hospitals Across Mexico from 1998 to 2017	Epidemiological	13.59	9.01	13.3	9.36	Yes
Helicobacter pylori and its association with autoimmune diseases: systemic lupus erythematosus, rheumatoid arthritis and Sjögren syndrome	Review	16.4	11.81	15.69	11.57	Yes
Achieving remission or low disease activity is associated with better outcomes in patients with systemic lupus erythematosus: a systematic literature review	Systematic Review	17.44	11.2	15.47	9.44	Yes
Bone mineral density and vertebral fractures in patients with systemic lupus erythematosus: A systematic review and meta-regression	Systematic Review	14.11	5.07	12.89	4.84	Yes
Factors Associated with Health-Related Quality of Life in Mexican Lupus Patients Using the LupusQol	Prospective	14.35	9.56	13.7	9.56	Yes
Mean (SD)		15.06 (1.68)	10.52 (2.17)	14.08 (1.21)	9.48 (1.86)	
*P value			<0.001		<0.001	

^a^ FKGL, Flesch-Kincaid grade level.

^b^ SMOG, simple measure of Gobbledygook; SD, standard deviation.

* *P*-values compare the readability scores of the original text vs. the ChatGPT-generated responses.

## Discussion

Overall, ChatGPT demonstrated a significant capacity (*P* < .001) to improve the accessibility of open-access, peer-reviewed scientific literature by reducing its average readability score by five grade points from the 15th to the 10th-grade level. Over the past year, coinciding with the emergence of LLMs exemplified by ChatGPT, there has been heightened attention on integrating these artificial intelligence models into healthcare and clinical education. This includes research into their use for developing patient-facing health content, assisting with postoperative patient management, and automating responses to typical questions (14, 15).

Drawing from our prior research, which has been among the initial investigations into the capacity of LLMs to process and present health-related information comprehensibly for patients, this study serves as a direct extension. Our foundational work, focusing on using models including GPT-3.5, GPT-4, and Copilot to address patient inquiries regarding antimalarial therapy in SLE (16), demonstrated high accuracy and reproducibility across models. Importantly, that study revealed significant variability in the completeness of responses, with GPT-4 exhibiting superior performance, particularly for complex topics like mechanism of action. Grounded on these findings, which highlighted both the potential and limitations of LLMs in delivering accurate and complete patient information, the present analysis evaluates explicitly the effectiveness of ChatGPT in a related but distinct application: simplifying the readability of complex peer-reviewed scientific literature within the broader area of rheumatology. This progression, from evaluating the content quality of direct answers to assessing the readability of simplified published research, reflects our ongoing commitment to exploring how AI can best enhance health literacy in rheumatologic care.

While our results demonstrate that ChatGPT can markedly improve the readability of complex rheumatology literature (reducing the average grade level from 15th grade to 10th grade), this simplified output remains well above recommended standards. The AMA and NIH advise that patient education materials be prepared around a sixth-grade reading level (2), far lower than the high school level achieved by ChatGPT in our study. This discrepancy is not a trivial gap; educational content pitched at a 10th-grade level still exceeds the literacy of many patients, undermining accessibility. Patients with limited health literacy—who often belong to disadvantaged groups—may struggle to comprehend information at this level, leaving them disadvantaged in managing their care and participating in informed decision-making. Other evaluations of ChatGPT's medical explanations have noted similar shortcomings, with average readability scores in the high school to college range (e.g., FKGL 13 in one study of fibromyalgia queries) (17). These findings raise important clinical and policy considerations. Clinically, suppose providers rely on ChatGPT to generate patient education handouts or summarize research. In that case, they must recognize that the “simplified” text may still be too complex for many readers, potentially perpetuating the communication barriers it aims to bridge. From a policy standpoint, there is a need for guidelines and oversight on using AI tools in patient education—ensuring that content is vetted or further refined to meet health literacy best practices (ideally at or below the 6th-grade level). Although iterative prompting or more advanced models might further lower the reading level towards compliance with guideline (18) healthcare professionals bear the final responsibility for confirming understandability and straightforwardness. Ultimately, the limitation is that ChatGPT's output does not yet consistently reach recommended readability levels, which is significant: addressing it is critical for promoting health equity and truly informed decision-making among patients with rheumatic diseases (8). As the literature emphasizes, improving readability is not merely an editorial concern but a moral imperative to ensure all patients—regardless of literacy—can access, understand, and use health information effectively (2, 19).

Our findings indicate that utilizing ChatGPT to simplify peer-reviewed rheumatology literature reduced readability from an average of 15th to 10th grade. This outcome contrasts with a similar study in the field of ophthalmology, where ChatGPT reduced the readability of articles from 15th grade to a 7th-grade level (20). One potential factor contributing to this difference could be the inherent complexity of rheumatic diseases, which often involve intricate pathological processes, diverse manifestations, and complex treatment regimens, potentially simplifying to a lower reading level more challenging than specific topics in ophthalmology.

Historically, the scientific literature's inherent complexity and technical language have posed significant barriers to patient comprehension. Prior research establishes a positive correlation between improved understanding of medical information and health literacy and outcomes such as increased trust in scientific bodies and enhanced patient capacity for self-management of health conditions (21, 22). Consequently, the advent and utilization of LLMs, exemplified by platforms like ChatGPT, present a potential mechanism for improving patient engagement with scientific content, possibly fostering a greater societal perception of the reliability of scientific research.

Patients could derive advantages from consulting peer-reviewed literature, predicated on the notion that enhanced research comprehension can positively influence multiple facets of their care (23). Extensive scholarship confirms that grasping the underlying justification for medical interventions significantly improves patient adherence to treatment protocols throughout diverse clinical fields (24). Strategies to mitigate the proliferation of health misinformation include enhancing health literacy, promoting collaborative use of online resources by patients and physicians, and establishing more robust indicators of source quality, as Swire-Thompson and Lazer described (25). Furthermore, direct public engagement with primary research outputs, including clinical trial results and peer-reviewed literature, potentially empowers patients toward more informed healthcare decision-making.

This study has limitations affecting its interpretation and application. Despite verification steps, the recognized propensity of ChatGPT to occasionally produce inaccurate information must be acknowledged (26). Thus, ChatGPT summaries of scientific literature should not serve as the sole basis for patient health education. Direct consultation with physicians concerning individual health conditions is strongly recommended. A second limitation involves the inherent nature of single studies; one article, viewed in isolation, may provide insufficient context or lack the comprehensive information necessary for sound healthcare decision-making. Therefore, patients seeking to make informed choices should consult various trusted information sources. Furthermore, it is currently unknown whether the accuracy or appropriateness of ChatGPT's responses could be negatively impacted by follow-up requests aimed at further shortening the generated text.

Third, although content readability is fundamental to comprehension, assessing the real-world impact of ChatGPT requires direct patient evaluation. Future prospective studies involving patient participants are necessary to determine the effectiveness of ChatGPT in enhancing the understanding of medical information and influencing health-related decision-making. Finally, the analysis was restricted to peer-reviewed scientific articles focused primarily on some rheumatic diseases, mainly SLE, and authored by the two senior investigators of this study. Consequently, the generalizability of these findings is limited, and caution is warranted against extrapolating the results to the broader fields of rheumatology.

In conclusion, this study demonstrates that the LLM ChatGPT 4.0 can significantly reduce the reading complexity of peer-reviewed rheumatology literature, transforming text requiring graduate-level reading proficiency to a more accessible 10th-grade level. Expert rheumatologists found the generated summaries to be accurate and appropriate, indicating the potential of LLMs as tools to bridge the communication gap between complex scientific findings and lay audiences. However, despite this marked improvement, the resulting readability still falls short of the recommended sixth-grade level for optimal patient education materials. This highlights a relevant limitation: while LLMs offer a promising avenue for enhancing health literacy, their current outputs may not be sufficiently simplified for all patients, particularly those with lower literacy levels. Therefore, while ChatGPT can be a valuable aid, its use in generating patient-facing summaries requires careful implementation, likely involving expert review and potential further refinement to meet established health literacy guidelines. Future research should focus on optimizing LLM prompts for greater simplification, evaluating the impact of these tools on actual patient comprehension and decision-making, and exploring their application across a broader range of rheumatologic topics to fully harness their potential while mitigating risks like inaccuracy or perpetuating health disparities.

## Data Availability

The raw data supporting the conclusions of this article will be made available by the authors, without undue reservation.

## References

[1] Benjamin RM. Improving health by improving health literacy. Public Health Rep. (2010) 125:784–5. 10.1177/003335491012500602 21121221 PMC2966655

[2] Rooney MK Santiago G Perni S Horowitz DP McCall AR Einstein AJ Readability of patient education materials from high-impact medical journals: a 20-year analysis. J Patient Exper. (2021) 8:2374373521998847. 10.1177/2374373521998847 34179407 PMC8205335

[3] Berkman ND Sheridan SL Donahue KE Halpern DJ Crotty K. Low health literacy and health outcomes: an updated systematic review. Ann Intern Med. (2011) 155:97–107. 10.7326/0003-4819-155-2-201107190-00005 21768583

[4] Milanti A Chan DNS Parut AA So WKW. Determinants and outcomes of eHealth literacy in healthy adults: a systematic review. PLoS One. (2023) 18(10):e0291229. 10.1371/journal.pone.0291229 37792773 PMC10550189

[5] Gorter A Bakker MM Ten Klooster PM Boonen A Vonkeman HE. The impact of health literacy: associations with disease activity and medication prescription in patients with rheumatoid arthritis. Rheumatology. (2023) 62:3409–15. 10.1093/rheumatology/kead094 36825825 PMC10547512

[6] Gong Z Haig SL Pope JE Rohekar S Rohekar G LeRiche NGH Health literacy rates in a population of patients with rheumatoid arthritis in Southwestern Ontario. J Rheumatol. (2015) 42:1610–5. 10.3899/jrheum.141509 26233510

[7] Maheswaranathan M Eudy AM Bailey SC Rogers JL Clowse ME. Low health numeracy is associated with higher disease activity in systemic lupus erythematosus. Lupus. (2021) 30:489–94. 10.1177/0961203320979044 33323013

[8] Bakker MM Putrik P Dikovec C Rademakers J Vonkeman HE Kok MR Exploring discordance between health literacy questionnaire scores of people with RMDs and assessment by treating health professionals. Rheumatology. (2022) 62:52–64. 10.1093/rheumatology/keac248 35438147 PMC9788830

[9] Hall AK Bernhardt JM Dodd V Vollrath MW. The digital health divide: evaluating online health information access and use among older adults. Heal Educ Behav. (2015) 42:202–9. 10.1177/1090198114547815 PMC4405138 25156311

[10] Kloosterboer A Yannuzzi NA Patel NA Kuriyan AE Sridhar J. Assessment of the quality, content, and readability of freely available online information for patients regarding diabetic retinopathy. JAMA Ophthalmol. (2019) 137:1240–5. 10.1001/jamaophthalmol.2019.3116 31436789 PMC6707011

[11] Plavén-Sigray P Matheson GJ Schiffler BC Thompson WH. The readability of scientific texts is decreasing over time. eLife. (2017) 6:e27725. 10.7554/eLife.27725 28873054 PMC5584989

[12] Fütterer T Fischer C Alekseeva A Chen X Tate T Warschauer M ChatGPT in education: global reactions to AI innovations. Sci Rep. (2023) 13:15310. 10.1038/s41598-023-42227-6 37714915 PMC10504368

[13] Zhang Y Wan X-H Kong Q-Z Liu H Liu J Guo J Evaluating large language models as patient education tools for inflammatory bowel disease: a comparative study. World J Gastroenterol. (2025) 31:102090. 10.3748/wjg.v31.i6.102090 39958450 PMC11752706

[14] Wong RS-Y Ming LC Raja Ali RA. The intersection of ChatGPT, clinical medicine, and medical education. JMIR Med Educ. (2023) 9:e47274. 10.2196/47274 37988149 PMC10698645

[15] Eysenbach G. The role of ChatGPT, generative language models, and artificial intelligence in medical education: a conversation with ChatGPT and a call for papers. JMIR Med Educ. (2023) 9:e46885. 10.2196/46885 36863937 PMC10028514

[16] Munguía-Realpozo P Mendoza-Pinto C Etchegaray-Morales I Méndez-Martínez S Ramírez-Lara E Solis-Poblano JC Evaluation of patient information provided by ChatGPT on antimalarial use in systemic lupus erythematosus: Spanish language translation. Int J Rheum Dis. (2025) 28:e70126. 10.1111/1756-185X.70126 39929781

[17] Uysal A Güntürk E. Quality and readability of ChatGPT’s responses to the most frequently searched words about fibromyalgia on google trends. Rheumatol Q. (2025) 3:12–9. 10.4274/qrheumatol.galenos.2025.07379

[18] Roster K Kann RB Farabi B Gronbeck C Brownstone N Lipner SR. Readability and health literacy scores for ChatGPT-generated dermatology public education materials: cross-sectional analysis of sunscreen and melanoma questions. JMIR Dermatol. (2024) 7:e50163. 10.2196/50163 38446502 PMC10955394

[19] Tucker CA. Promoting personal health literacy through readability, understandability, and actionability of online patient education materials. J Am Heart Assoc. (2024) 13:e033916. 10.1161/JAHA.124.033916 38567677 PMC11262515

[20] Kianian R Sun D Rojas-Carabali W Agrawal R Tsui E. Large language models may help patients understand peer-reviewed scientific articles about ophthalmology: development and usability study. J Med Internet Res. (2024) 26:e59843. 10.2196/59843 39719077 PMC11707445

[21] Chen X Hay JL Waters EA Kiviniemi MT Biddle C Schofield E Health literacy and use and trust in health information. J Health Commun. (2018) 23:724–34. 10.1080/10810730.2018.1511658 30160641 PMC6295319

[22] Tsai T-I Yu W-R Lee S-YD. Is health literacy associated with greater medical care trust? Int J Qual Health Care. (2018) 30:514–9. 10.1093/intqhc/mzy043 29608676

[23] Murphy CK. Do patients need to read research? Knowledge is power. Br Med J. (2003) 327:564. 10.1136/bmj.327.7414.564-b PMC192871 12958130

[24] Miller TA. Health literacy and adherence to medical treatment in chronic and acute illness: a meta-analysis. Patient Educ Couns. (2016) 99:1079–86. 10.1016/j.pec.2016.01.020 26899632 PMC4912447

[25] Swire-Thompson B Lazer D. Public health and online misinformation: challenges and recommendations. Annu Rev Public Health. (2020) 41:433–51. 10.1146/annurev-publhealth-040119-094127 31874069

[26] Emsley R. ChatGPT: these are not hallucinations—they’re fabrications and falsifications. Schizophr. (2023) 9:52. 10.1038/s41537-023-00379-4 PMC10439949 37598184

